# Confidence and uncertainty aware deep learning for reliable grape leaf disease diagnosis under real world field conditions

**DOI:** 10.3389/fpls.2026.1839652

**Published:** 2026-07-16

**Authors:** Ebru Ergün, Hatice Okumus, Omer Emin Cinar, Nevzat Batan

**Affiliations:** 1 Department of Electrical and Electronics Engineering, Faculty of Engineering and Architecture, Recep Tayyip Erdogan University, Rize, Türkiye; 2 Department of Electrical and Electronics Engineering, Faculty of Engineering, Karadeniz Technical University, Trabzon, Türkiye; 3 Department of Computer Engineering, Faculty of Engineering and Architecture, Recep Tayyip Erdogan University, Rize, Türkiye; 4 Department of Molecular Biology and Genetics, Faculty of Science, Karadeniz Technical University, Trabzon, Türkiye

**Keywords:** confidence calibration, deep learning, field conditions, grape leaf disease, precision agriculture, uncertainty estimation

## Abstract

Accurate and reliable diagnosis of grape leaf diseases is essential for sustainable viticulture, enabling timely intervention, reducing economic losses, and supporting precision crop management. Although deep learning (DL) models have demonstrated remarkable classification performance, their reliability under real-world field conditions remains insufficiently explored. In particular, confidence estimates often fail to reflect true predictive correctness when models are exposed to distributional shifts, limiting their practical applicability. To address this challenge, this study proposes a confidence- and uncertainty-aware DL framework for grape leaf disease diagnosis that extends evaluation beyond conventional accuracy-based metrics. Two publicly available grape leaf datasets were employed for stratified five-fold cross-validation in binary and multiclass classification tasks, while a third independently collected dataset was reserved exclusively for leakage-free external validation. EfficientNet-B0 and MobileNetV3-Large were evaluated under four inference configurations: raw inference, temperature scaling (TempScaling), Monte Carlo dropout, and an ensemble strategy combining uncertainty estimation with calibration. External validation demonstrated that both architectures maintained discriminative capability under domain shift. Under ensemble inference, EfficientNet-B0 achieved an accuracy of 73.8%, a macro-F1 score of 73.3%, a Matthews correlation coefficient (MCC) of 0.468, and a receiver operating characteristic area under the curve (ROC-AUC) of 0.804, while MobileNetV3-Large achieved 71.8% accuracy, 71.2% macro-F1, an MCC of 0.429, and a ROC-AUC of 0.787. Despite these promising results, raw predictions exhibited substantial overconfidence, with Expected Calibration Error (ECE) values of 0.287 and 0.312 for EfficientNet-B0 and MobileNetV3-Large, respectively. TempScaling markedly improved calibration quality, reducing ECE to 0.038 and 0.042 without affecting classification performance. Ensemble inference further enhanced the balance between predictive discrimination and reliability. The results demonstrate that strong classification performance alone is insufficient for trustworthy deployment in agricultural environments. Confidence calibration and uncertainty quantification provide complementary information for identifying overconfident predictions and improving decision reliability under field variability. The proposed framework offers a reliability-oriented approach for developing trustworthy artificial intelligence systems for grape leaf disease diagnosis in precision agriculture.

## 1 Introduction

### 1.1 Background

Viticulture faces persistent yield and quality losses from foliar diseases that alter canopy function, reduce photosynthetic capacity, and increase production costs through repeated monitoring and intervention (Chen et al., 2025; Barbedo, 2018). In commercial vineyards, decisions are often taken under time pressure using images captured with handheld devices in heterogeneous illumination, complex backgrounds, and variable symptom severity. Under these conditions, a diagnostic tool must do more than assign a disease label; it also needs to convey whether its prediction is reliable enough to support an action, because overconfident errors could delay control measures or trigger unnecessary treatments (Mohanty et al., 2016; Ferentinos, 2018).

Early grape disease studies relied on handcrafted image processing pipelines aimed at segmenting symptomatic regions and classifying diseases using conventional learners. Meunkaewjinda et al. (2008) developed a multi-stage pipeline for color and disease segmentation using self-organizing maps and support vector machines (SVM) to categorize scab, rust, and healthy cases. Sannakki et al. (2013) processed field-like images by removing noise via anisotropic diffusion and segmenting regions through K-means clustering, emphasizing the risk of inappropriate pesticide use when severity was misjudged. Similarly, Padol and Yadav (2016) used K-means and SVM for classification, reporting moderate accuracy.

With the rise of deep learning (DL), research shifted toward end-to-end recognition models. Nagi and Tripathy (2022) designed a lightweight six-layer CNN, while Sasikaladevi (2025) adopted transfer learning with EfficientNet-B0, reporting high validation accuracy and Receiver operating characteristic area under the curve (ROC-AUC) analysis. Nasra and Gupta (2024) fine-tuned Xception-based architectures, and Sood and Singh (2024) introduced Gaussian noise during training to reduce overfitting. Kunduracioglu and Pacal (2024) benchmarked various CNN and vision transformer models, reporting extremely high accuracies. Further modular pipelines and specialized pixel-encoding methods have been proposed to improve discrimination on curated datasets like PlantVillage (Shantkumari and Uma, 2023a, Shantkumari and Uma, 2023b; Talaat et al., 2025).

### 1.2 Gaps

Despite these advancements, a significant gap exists regarding model reliability in the face of “domain shift”, the transition from curated training data to the unpredictability of the vineyard. Many existing frameworks demonstrate high discrimination on benchmark sets like PlantVillage but lack proven robustness in unseen field scenes (Shantkumari and Uma, 2023a, 2023b; Talaat et al., 2025). Various strategies have attempted to bridge this gap: Jin et al. (2022) used GAN-based augmentation to combat data scarcity, while Zhu et al. (2021) applied super-resolution to YOLOv3 to detect small lesions, noting that performance still declined as background complexity increased. Others have focused on deployment constraints, such as Zinonos et al. (2021), who integrated LoRa communication with Grad-CAM for low-resolution interpretability, and Adão et al. (2025), who utilized diffusion-based synthetic data to accelerate training. More recently, Li et al. (2025) proposed multimodal transformers to fuse RGB and environmental data, and Radhey et al. (2024) highlighted the importance of IoT ecosystems for timely intervention.

However, these advancements largely overlook the central question of reliability: whether a model’s probability score truly reflects its likelihood of correctness under domain shift. Modern networks are often “confidently wrong,” yet calibration metrics such as Expected Calibration Error (ECE) and Negative Log-Likelihood (NLL) are rarely reported in grape disease literature. This issue has recently attracted increasing attention in the broader artificial intelligence community. Dussert et al. (2025) demonstrated that DL models may exhibit substantial confidence miscalibration despite strong classification performance and showed that temperature scaling (TempScaling) can effectively improve the reliability of confidence estimates without affecting predictive outcomes. Similarly, Dayal et al. (2025) employed a Bayesian DL framework for agricultural prediction tasks and reported that explicitly modelling uncertainty significantly improved predictive robustness and reduced estimation error under complex environmental conditions. Collectively, these studies highlight the importance of calibration- and uncertainty-aware evaluation and suggest that reliability-oriented assessment should complement conventional accuracy-based performance analysis in real-world agricultural applications.

### 1.3 Contributions

This study addresses these shortcomings by introducing a reliability-focused DL framework for grape leaf diagnosis. The primary contributions are:

- Strict External Validation: Utilizing two public field datasets for training while reserving an entirely independent third dataset for external evaluation to measure the impact of domain shift.
- Uncertainty-Aware Inference: Evaluating multiple strategies to improve prediction trustworthiness, including TempScaling, Monte Carlo dropout (MCDropout), and ensemble methods.
- Calibration Quantification: Shifting the focus from simple accuracy to actionable reliability by assessing how well a model’s confidence aligns with its actual field performance.
- Efficiency and Deployment: Employing lightweight backbones to ensure the framework remains computationally viable for real-world viticulture applications.

### 1.4 Paper organization

The remainder of this article is structured as follows. Section 2 describes the datasets employed in the study and presents the proposed research methodology, including the model backbones, training protocol, cross-validation strategy, uncertainty-aware inference methods, and evaluation metrics. Section 3 reports the experimental results, including classification performance, calibration analysis, and external validation findings. Section 4 discusses the obtained results, examines the reliability of DL models under real-world field conditions, and highlights the implications of confidence calibration and uncertainty quantification for practical agricultural applications. Finally, Section 5 concludes the study by summarizing the main findings, contributions, limitations, and potential directions for future research.

## 2 Materials and methods

### 2.1 Description of datasets

#### 2.1.1 Dataset1

**Figure 1:**
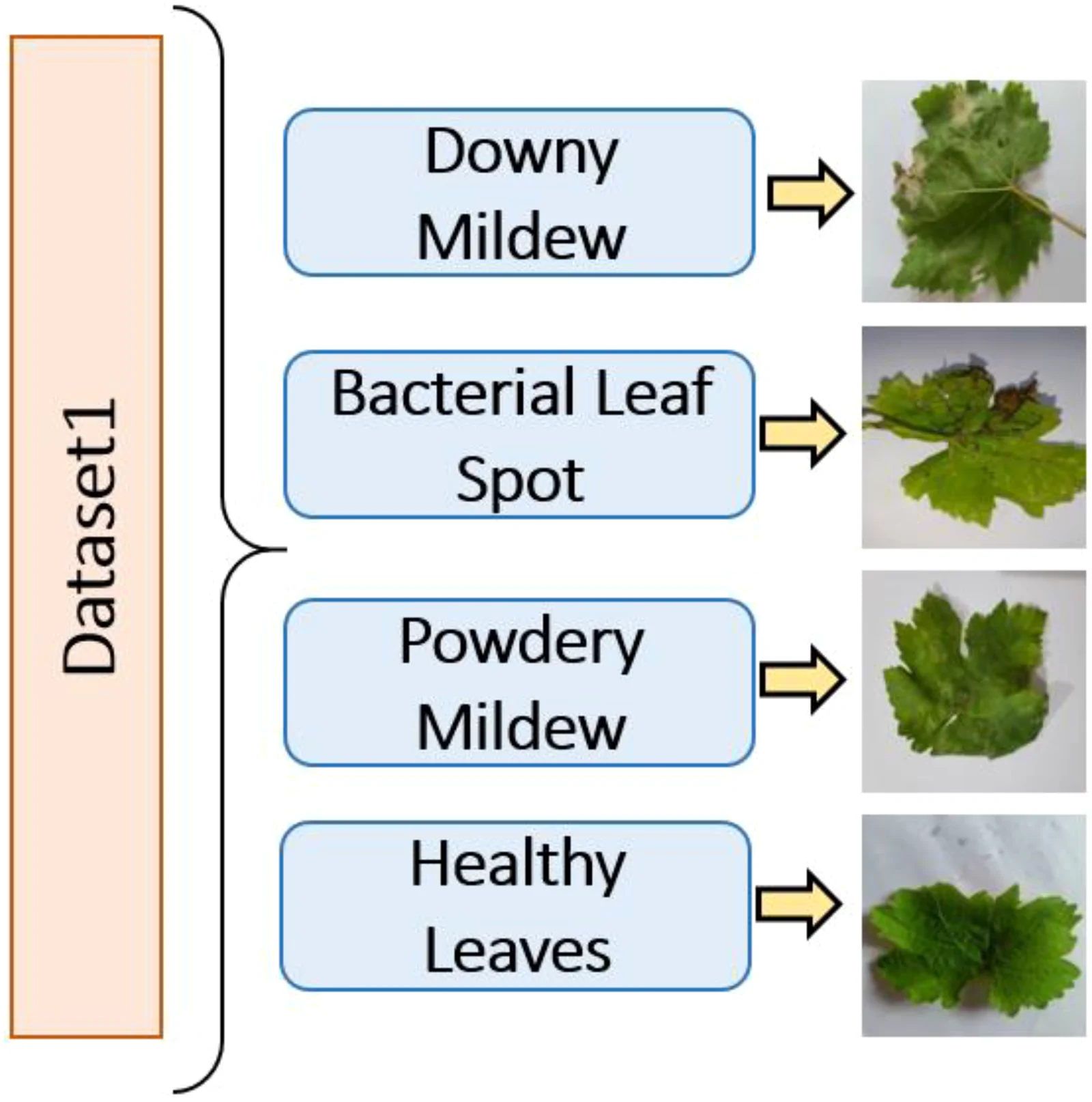
Representative sample images of grape leaves from Dataset1, illustrating healthy leaves and different disease categories (Dharrao et al., 2025).

The first dataset employed in this study was publicly released by Dharrao et al. (2025) and was selected due to its close alignment with real-world vineyard conditions. The dataset consists of 2,726 high-quality RGB images of table grape leaves acquired directly in natural field environments, which is critical for assessing model generalization beyond laboratory settings. The images are grouped into four classes representing common grapevine health conditions: downy mildew, bacterial leaf spot, powdery mildew, and healthy leaves. Each class is organized within an independent directory structure to facilitate supervised learning. Image acquisition was performed using mobile devices under uncontrolled illumination and background conditions, thereby preserving environmental variability. All images were standardized to a spatial resolution of 256 × 256 pixels with a density of 96 dpi. Representative samples from each disease category are illustrated in **Figure 1**.

#### 2.1.2 Dataset2

**Figure 2:**
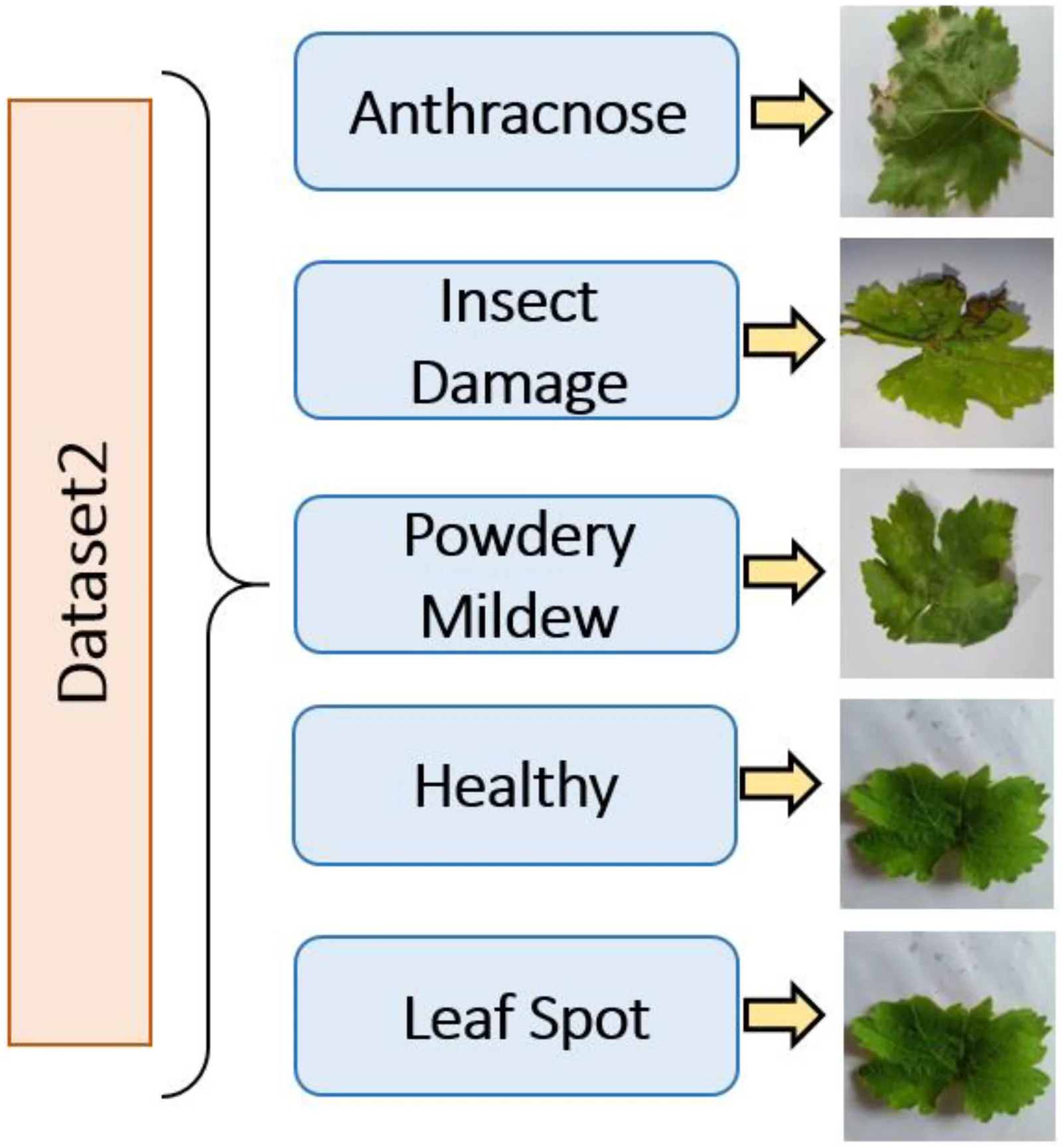
Representative sample images of grape leaves from Dataset2, covering healthy leaves and multiple disease conditions (Rahman et al., 2025).

The second dataset utilized in this study was publicly released by Rahman et al. (2025) and is referred to as the Burmese grape leaf disease dataset. This dataset comprises a total of 3,103 high-quality RGB images of grapevine leaves collected under real field conditions. It was designed to represent diverse health states of grape leaves and includes five distinct classes: healthy leaves (1,006 images), anthracnose or brown spot disease (447 images), insect damage (990 images), powdery mildew (296 images), and yellow leaf spot (364 images). The class distribution reflects realistic disease prevalence patterns observed in vineyard environments. All images were captured directly in natural outdoor settings using two different mobile devices, namely Realme 8 and Redmi Note 7 Pro Max smartphones, ensuring variability in illumination, background complexity, and acquisition conditions. The dataset was collected at multiple vineyard locations, further enhancing environmental diversity and reducing spatial bias. Each image was selected to exhibit clear and distinguishable visual symptoms associated with the corresponding leaf condition, facilitating reliable feature learning for automated disease recognition. To improve data diversity and mitigate class imbalance, the dataset creators additionally provided an augmented version generated through controlled image transformations, including brightness and contrast adjustments, geometric rotations, shear operations, and zoom-based scaling. However, in this study, only the original, non-augmented images were employed to preserve the natural distribution of field-acquired data and to ensure a fair evaluation of model generalization. Representative samples from each disease category are presented in **Figure 2**.

#### 2.1.3 Dataset3

The third dataset was constructed through a dedicated field-based image acquisition campaign targeting grape leaf diseases commonly observed in vineyards of the Black Sea region of Türkiye. The dataset was specifically designed to capture regional variability and naturally occurring disease symptoms under real cultivation conditions, thereby enabling a realistic and unbiased evaluation of model performance in practical deployment scenarios. A multi-site survey was conducted across four vineyards, including three located in Trabzon and one in Rize. Within each vineyard, 30 grapevines were randomly selected, and five leaves were sampled from each vine to represent different spatial positions across the canopy. This standardized sampling protocol resulted in 600 initial *in situ* leaf observations. After visual inspection, quality control, and exclusion of ambiguous or low-quality samples, the curated Dataset3 was structured as a binary classification dataset.

**Figure 3:**
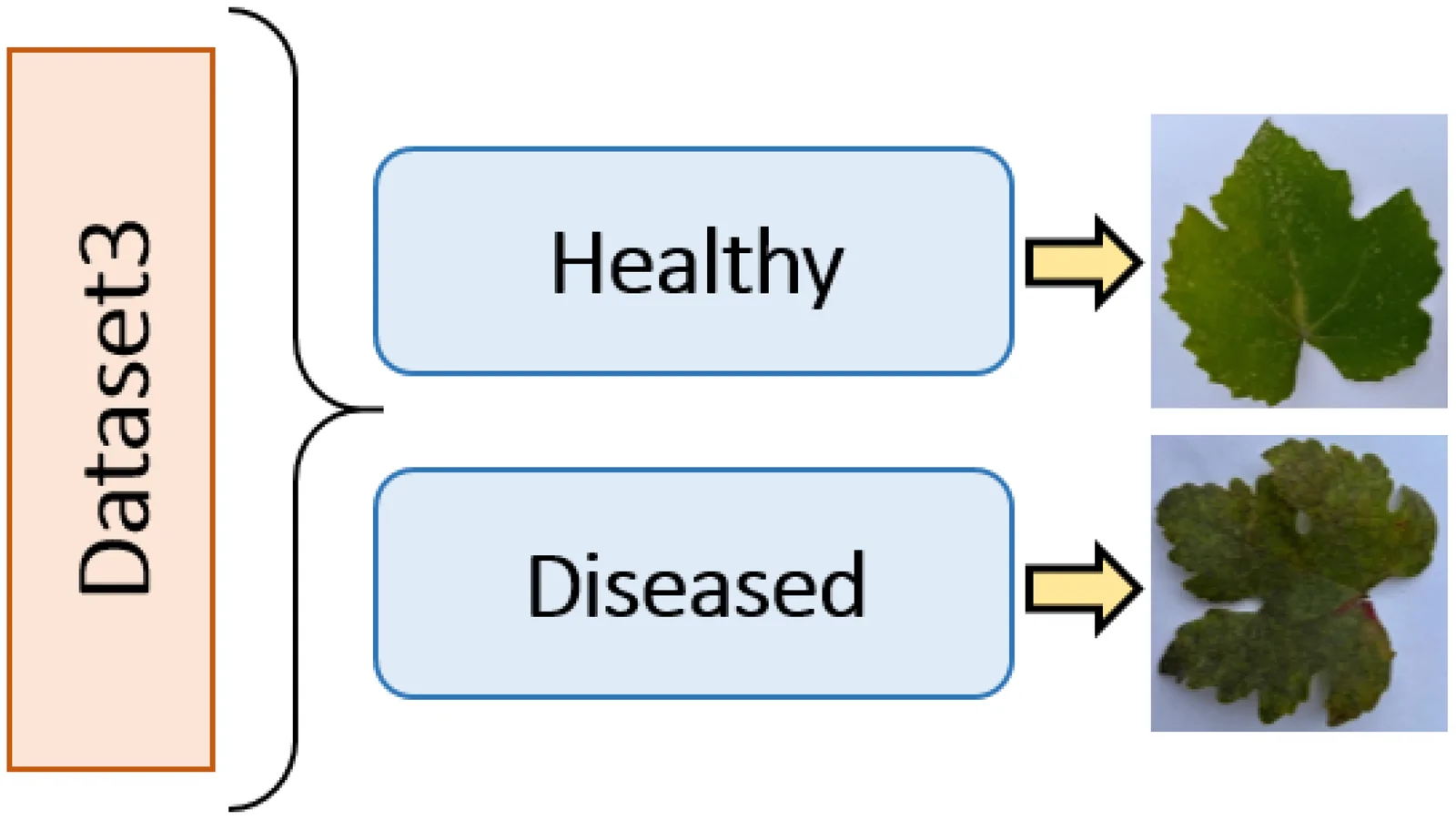
Representative sample images of grape leaves from Dataset3.

Following field inspection, leaves exhibiting clearly visible disease symptoms or healthy morphology were selected for imaging. Image acquisition was performed under natural lighting conditions using an iPhone 14 Pro Max, ensuring high-resolution imagery with accurate color fidelity. Raw images were recorded at a minimum resolution of 1024 × 1024 pixels and subsequently resized to 256 × 256 pixels prior to being used as inputs for the DL models. Annotation of the collected images was performed based on the visual symptoms observed directly in the field during the acquisition stage. Leaves showing clear pathological symptoms such as chlorotic regions, necrotic lesions, powdery fungal structures, or abnormal discoloration patterns were labeled as diseased, while leaves without visible symptoms were labeled as healthy. To ensure labeling consistency, only samples exhibiting visually distinguishable and well-defined symptoms were retained in the dataset, while ambiguous or low-quality images were excluded during the data curation process. To enhance the robustness of the external validation protocol and increase the representativeness of the field dataset, an additional collection phase was conducted under the same acquisition, annotation, and quality-control procedures described above. During this second collection campaign, 50 additional healthy leaf images and 50 additional diseased leaf images were acquired and incorporated into Dataset3. The final dataset was structured as a binary classification problem comprising two classes: healthy leaves and diseased leaves. Consequently, Dataset3 consisted of 300 healthy images and 200 diseased images, yielding a total of 500 labeled samples. Representative examples from each class are presented in **Figure 3**.

All images in Dataset3 were collected independently of the public datasets used for model development and were excluded from all training, hyperparameter optimization, model selection, and calibration procedures. Consequently, Dataset3 served exclusively as a leakage-free external test set for evaluating discrimination performance, confidence calibration, and uncertainty behavior under real-world field conditions. Access to vineyard areas and image acquisition activities were conducted with the permission of vineyard owners. The study did not involve human participants, personal data, or protected biological materials.

### 2.2 Research methodology framework

**Figure 4:**
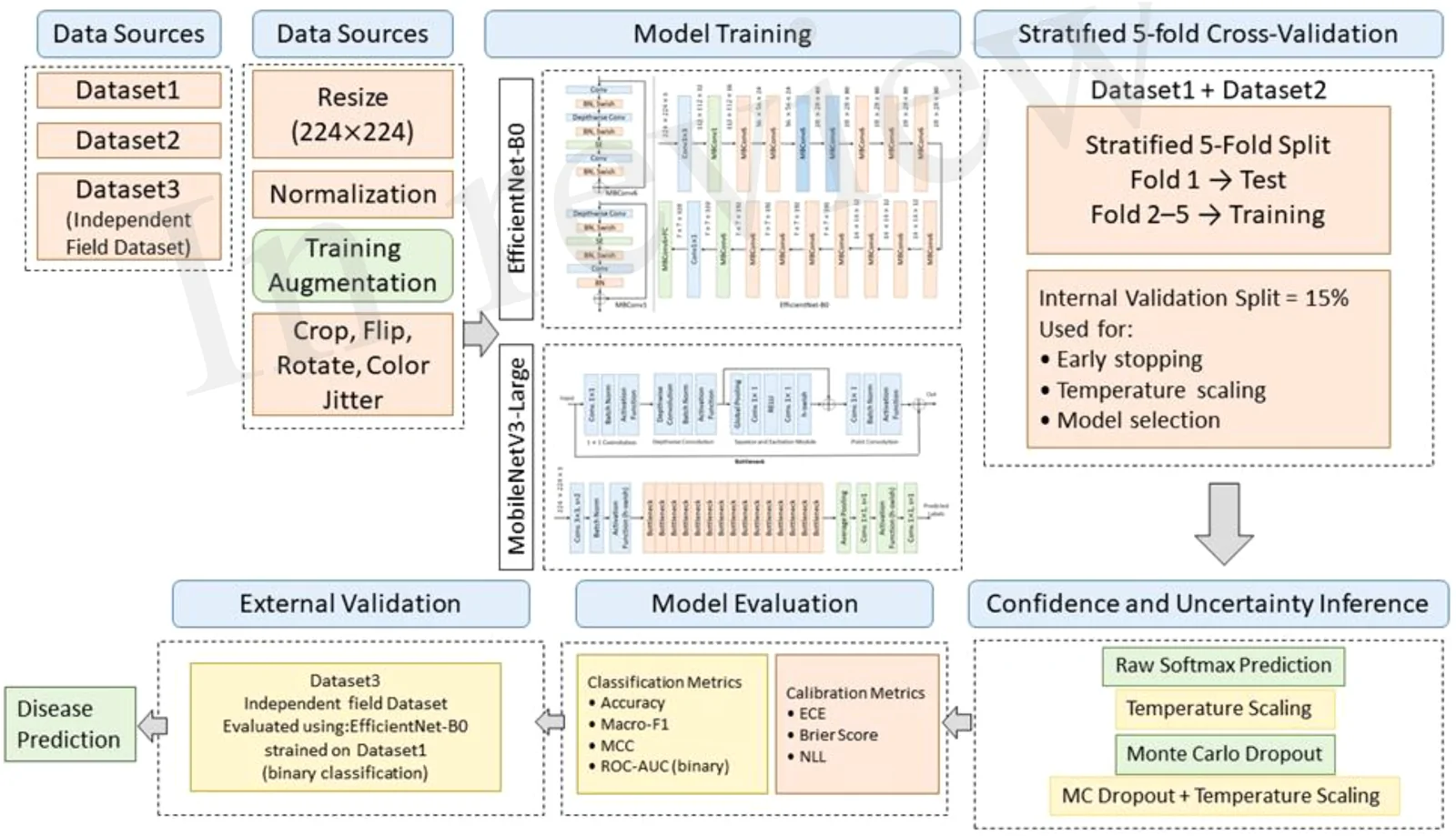
Research methodology framework of the proposed confidence- and uncertainty-aware grape leaf disease diagnosis pipeline.

This study follows a structured experimental framework that integrates dataset preparation, model training, uncertainty-aware inference, and reliability-focused evaluation into a unified pipeline. The methodological flow, illustrated in **Figure 4**, was designed to assess not only classification performance but also the consistency and calibration of predictive confidence under varying data conditions. Three datasets with complementary characteristics were incorporated into the experimental design. Two publicly available datasets were used for model training and validation through stratified cross-validation to ensure balanced class representation across folds. An additional dataset, independently collected and excluded from the training process, was reserved solely for external evaluation. External validation was conducted using fold-specific EfficientNet-B0 and MobileNetV3-Large checkpoints obtained from the Dataset1 binary classification experiments. This separation enabled an unbiased examination of generalization behavior under distribution shifts commonly encountered in real field environments.

All images were resized to 224 ×224 pixels before being provided to the networks. Input normalization was performed using the ImageNet mean and standard deviation values (mean = [0.485, 0.456, 0.406], standard deviation = [0.229, 0.224, 0.225]) to ensure compatibility with the pretrained backbone weights. During training, online data augmentation was applied to improve robustness against natural variability in field-acquired imagery. The augmentation pipeline included random resized cropping with a scale range of 0.90–1.00, random horizontal flipping with a probability of 0.5, random rotation up to 15°, and mild color jittering in brightness, contrast, saturation, and hue. For validation and test subsets, only deterministic resizing and normalization were applied to preserve evaluation consistency.

Each dataset was processed independently to preserve its inherent distributional properties. Model training was conducted using EfficientNet-B0 and MobileNetV3-Large, two lightweight convolutional architectures selected for their favorable trade-off between predictive performance, computational efficiency, and deployment suitability. A stratified five-fold cross-validation (5-FCV) protocol was adopted, and within each fold an internal validation subset was extracted from the training data to guide early stopping and calibration procedures. Optimization was performed using the AdamW optimizer with explicit weight decay, while early stopping was employed to mitigate overfitting and stabilize learning across folds. Following training, inference was extended beyond conventional softmax-based prediction through multiple uncertainty-aware strategies. Baseline confidence estimates were obtained using raw softmax probabilities. TempScaling was subsequently applied, where a scalar temperature parameter was optimized on validation logits and applied at test time to recalibrate predicted probabilities. Epistemic uncertainty was further explored through MCDropout by enabling stochastic forward passes during inference, while batch normalization layers were kept fixed to maintain consistent feature distributions. For all MCDropout experiments, M = 20 stochastic forward passes were performed, and the resulting predictive distributions were averaged to estimate model uncertainty. A combined inference strategy based on Monte Carlo-averaged logits and TempScaling was additionally used to jointly address uncertainty estimation and probability calibration. Model evaluation encompassed both discriminative performance and calibration quality. Standard classification metrics, including accuracy and macro-averaged F1 score, were reported alongside calibration-oriented measures such as ECE, Brier score, and NLL. Additional analyses, including reliability diagrams, confidence histograms, confusion matrices, and ROC-AUC curves for binary classification tasks, were used to provide a comprehensive assessment of predictive reliability. To further evaluate the robustness of the reliability estimates, Wilcoxon signed-rank tests were performed on fold-wise ECE and NLL values, and bootstrap-based 95% confidence intervals were computed for external validation metrics obtained on Dataset3.

For the binary classification setting, all non-healthy categories in Dataset1 and Dataset2 were merged into a single diseased class, while healthy leaves were preserved as the second class. Accordingly, the insect damage category in Dataset2 was also included in the diseased group for binary experiments.

### 2.3 Model backbones and training Protocol

In this study, the model design and training strategy were structured to balance discriminative performance, computational efficiency, and deployment feasibility under real-world field conditions. Rather than prioritizing large-scale architectures optimized solely for benchmark accuracy, the framework emphasized lightweight convolutional backbones combined with a carefully controlled training protocol. This design choice aimed to ensure robustness against domain shifts while maintaining practical applicability for precision agriculture scenarios, where computational resources and inference latency are often critical. Two lightweight DL architectures, EfficientNet-B0 and MobileNetV3-Large, were employed as backbone models. Both networks were initialized with ImageNet-pretrained weights to leverage transferable low-level and mid-level visual representations learned from large-scale natural image datasets. For each backbone, the final classification layer was replaced with a task-specific fully connected layer corresponding to the number of target classes in the dataset. All remaining layers were fine-tuned during training without freezing any backbone parameters, enabling full adaptation of pretrained features to grape leaf disease characteristics.

The experimental pipeline was implemented using the PyTorch DL framework. To improve reproducibility across folds and experimental runs, random seeds were fixed across Python, NumPy, and PyTorch operations, and deterministic CuDNN behavior was enforced during training. All experiments were conducted using a batch size of 16. Model optimization was performed using the AdamW optimizer with a learning rate of 1 × 10–^4^ and a weight decay coefficient of 1 × 10^–4^. The cross-entropy loss function was employed for all classification tasks. Early stopping was applied by monitoring validation accuracy, and training was terminated when no improvement was observed for seven consecutive epochs. The maximum number of epochs was set to 30 for Dataset1 and binary classification experiments, and 35 for Dataset2 due to its larger size and higher-class diversity.

To obtain reliable performance estimates and reduce bias associated with a single train–test split, a stratified (5-FCV) protocol was adopted. Stratification ensured that the class distribution remained consistent across all folds. In each iteration, one fold was reserved as the test subset, while the remaining four folds were used for model development. From this development subset, 15% of the samples were further separated as an internal validation set using stratified sampling. This validation set was used exclusively for early stopping, model selection, and TempScaling parameter estimation. The held-out test fold remained completely unseen during both optimization and calibration stages, ensuring unbiased fold-wise evaluation. The training and evaluation pipeline was implemented using dedicated data loaders with parallel data loading workers, and model checkpoints were saved for the best validation-performing epoch within each fold. This configuration ensured stable optimization behavior and consistent experimental reproducibility across all datasets and backbone architectures.

#### 2.3.1 EfficientNet-B0

**Figure 5:**
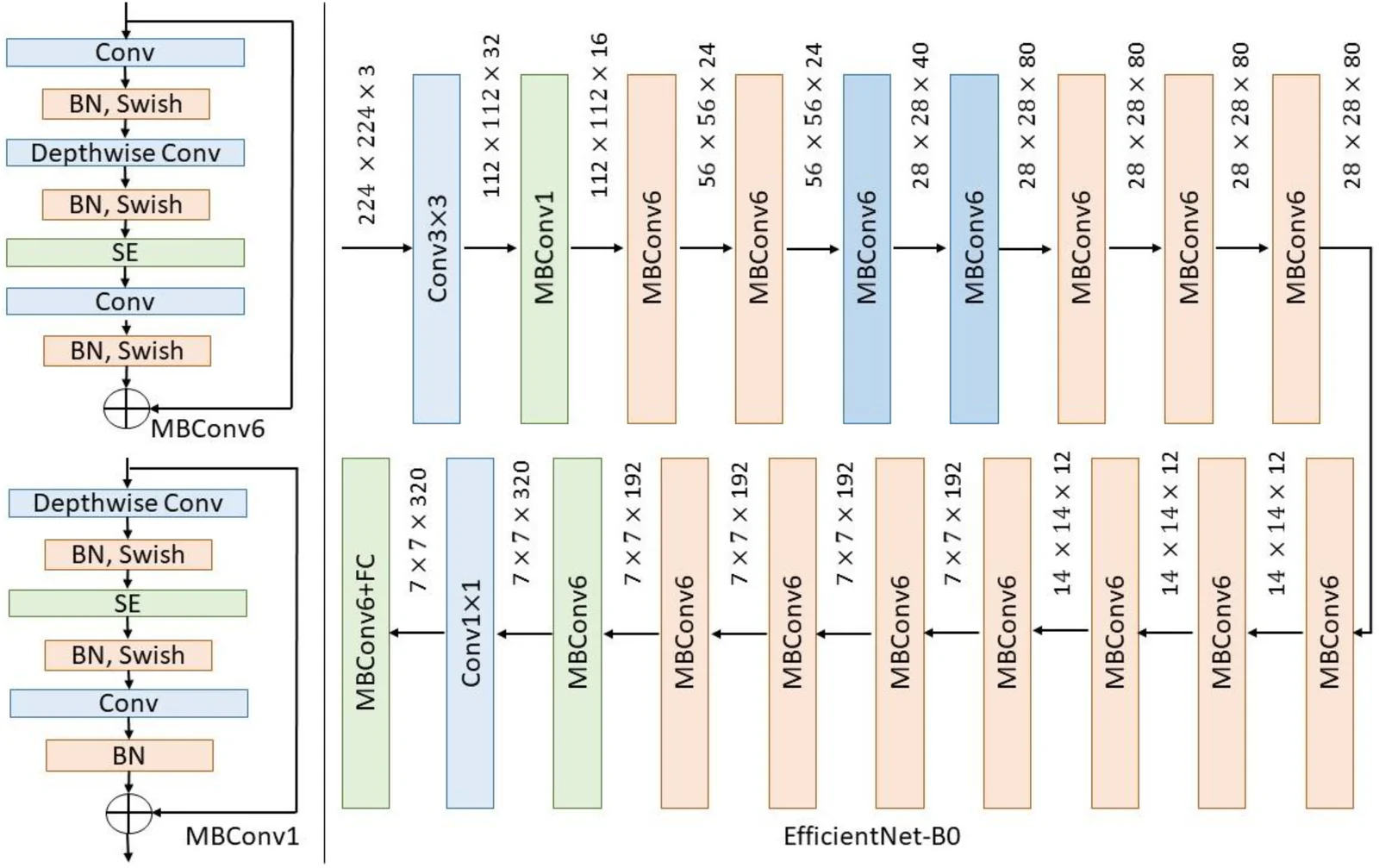
Architectural overview of the EfficientNet-B0 backbone employed in this study, illustrating the compound-scaled network structure composed of MBConv blocks with SE attention, followed by a task-specific classification head.

EfficientNet-B0 was adopted as a primary backbone due to its compound scaling strategy, which systematically balances network depth, width, and input resolution under a unified optimization framework (Ali et al., 2025; Singh et al., 2025). Unlike conventional convolutional neural networks (CNN) that expand these dimensions independently, EfficientNet introduces a coordinated scaling mechanism that preserves representational efficiency while constraining computational cost. The core design principle of EfficientNet is based on compound scaling, where model capacity is increased using a single scaling coefficient ϕ. Depth d, width w, and resolution r are jointly scaled according to Equation 1. The depth, width, and resolution dimensions are jointly scaled according to Equation 1, subject to the computational constraint given in Equation 2.

(1) $$d=\alpha^{\emptyset },\;\;w=\beta^{\emptyset },\;\;\;\;\;\;\;\;\;r=\gamma^{\emptyset }$$

(2) $$a\cdot \;\;\beta^{2}\cdot \;\gamma^{2}\approx 2$$

where α, β, and γ are constants determined via grid search under a fixed computational budget. Equations 1 and 2 ensure that model scaling remains computationally feasible while improving feature learning capacity in a balanced manner. As illustrated in **Figure 5**, EfficientNet-B0 is composed of a sequence of mobile inverted bottleneck convolution (MBConv) blocks augmented with squeeze-and-excitation (SE) attention modules. The MBConv structure employs depthwise separable convolutions to reduce parameter count, while the SE mechanism adaptively recalibrates channel-wise feature responses (Ali et al., 2025). This architectural combination enables the network to emphasize disease-relevant visual cues, such as lesion texture and chromatic irregularities, while suppressing background noise commonly present in field-acquired grape leaf images. In the proposed framework, the final classification layer of EfficientNet-B0 was replaced with a task-specific fully connected layer matching the number of grape leaf disease classes. All remaining layers were initialized with ImageNet-pretrained weights, allowing the model to leverage transferable low-level visual representations while adapting higher-level features to the target domain.

#### 3.1.2 MobileNetV3-large

**Figure 6:**
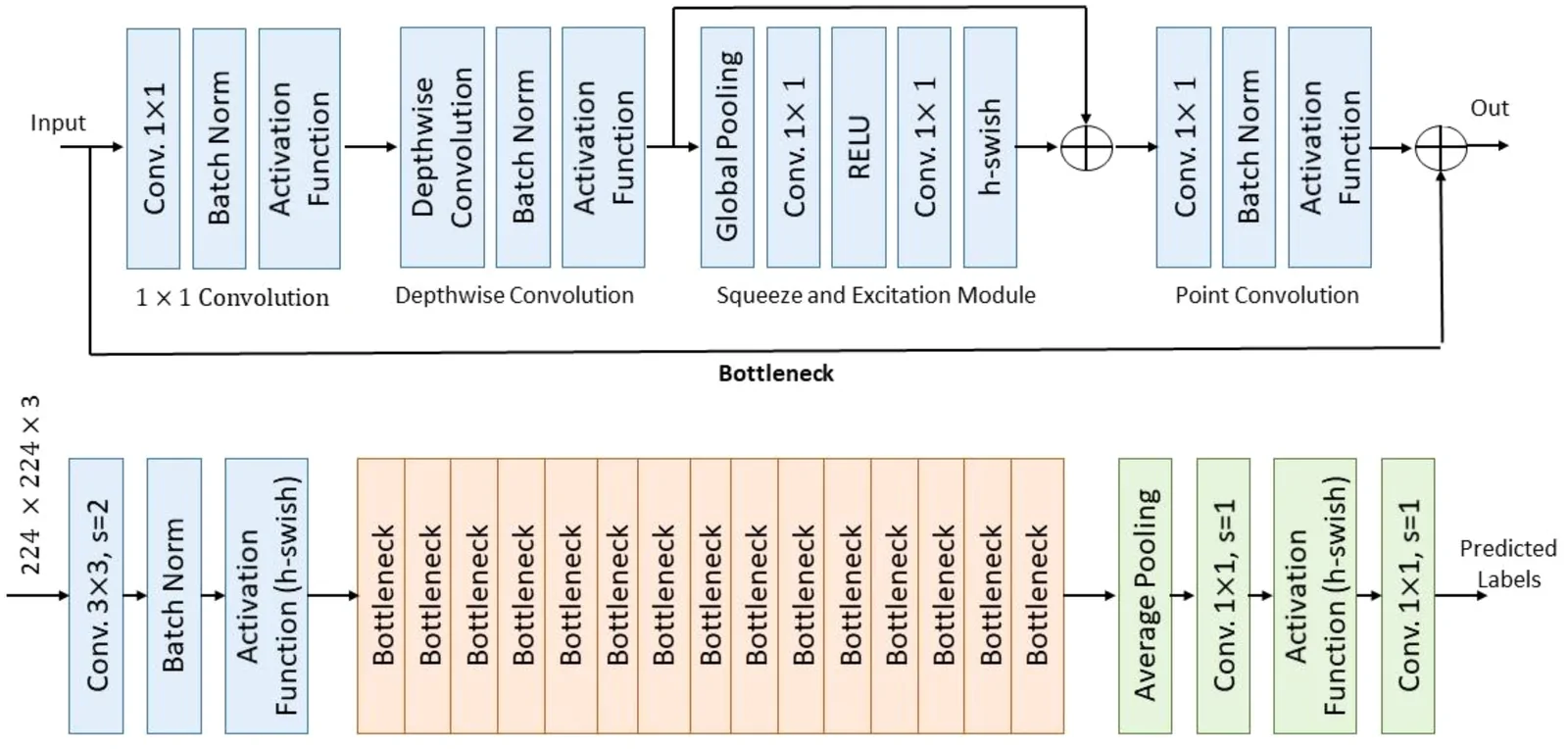
Structural representation of the MobileNetV3-Large backbone, highlighting depthwise separable convolutions, inverted residual blocks, SE modules, and the h-swish activation function used for efficient feature extraction.

MobileNetV3-Large was selected as a complementary lightweight backbone to investigate uncertainty-aware behavior across architectures optimized for mobile and edge deployment (Maximilliano and Rachmat, 2025; Zhang et al., 2025). The model integrates depthwise separable convolutions, lightweight attention mechanisms, and activation functions optimized for low-precision inference. A key architectural component of MobileNetV3-Large is the use of inverted residual blocks combined with linear bottlenecks. Given an input feature map X, depthwise separable convolution decomposes standard convolution into depthwise and pointwise operations, which can be expressed as Equation 3;

(3) $$Y=(X*K_{d})*K_{p}$$

where $K_{d}$​ denotes the depthwise convolution kernel applied independently to each channel, and $K_{p}$​ represents the pointwise 1 ×1 convolution used for channel mixing. Equation 3 highlights how computational complexity is significantly reduced compared to standard convolution, making the architecture suitable for resource-constrained environments. MobileNetV3-Large further incorporates channel-wise attention through lightweight SE blocks, allowing the network to dynamically prioritize informative feature channels (Abiyyu and Rahardi, 2026). Additionally, the architecture employs the h-swish activation function, defined as Equation 4.

(4) $$\text{h}-\text{swish}(\text{x})=\text{x}\cdot \frac{ReLU6(x+3)}{6}$$

which approximates the swish function while maintaining computational efficiency. This activation function improves non-linear representational capacity without incurring excessive inference cost, as shown in Equation 4. The overall structure of MobileNetV3-Large, depicted in **Figure 6**, consists of stacked inverted residual blocks with varying expansion factors, followed by a lightweight classification head. Similar to EfficientNet-B0, the final classifier layer was adapted to the grape leaf disease classes, while pretrained weights were used for initialization.

### 3.2 Optimization and early stopping

Model optimization was carried out using the AdamW optimizer, which decouples weight decay from the gradient-based parameter update, thereby providing improved generalization behavior compared to conventional adaptive optimization schemes. Given a parameter vector θ, AdamW updates were computed as Equation 5 (Zhou et al., 2024);

(5) $$\theta_{t+1}=\theta_{t}-\eta \cdot \frac{{\hat{m}}_{t}}{\sqrt{{\hat{v}}_{t}+\epsilon }}-\eta \cdot \lambda \cdot \theta_{t}$$

where η denotes the learning rate, λ represents the weight decay coefficient, and ${\hat{m}}_{t}$ and ${\hat{v}}_{t}$​ correspond to bias-corrected first- and second-order moment estimates, respectively. The explicit regularization term in Equation 5 ensured controlled parameter growth and mitigated overfitting, particularly under limited and imbalanced training data conditions. A fixed learning rate of 1 $\times 10^{-4}$ was employed across all experiments to maintain stable convergence behavior. Weight decay was set to 1 $\times 10^{-4}$, balancing regularization strength without suppressing feature learning. Early stopping was introduced as an additional regularization mechanism, where training was terminated if the validation accuracy failed to improve for a predefined patience window of seven consecutive epochs. This strategy prevented unnecessary training cycles and reduced the risk of overfitting to fold-specific data distributions. Epoch configurations were adjusted according to dataset characteristics. For Dataset1, training was conducted for a maximum of 30 epochs, whereas Dataset2 was trained for up to 35 epochs due to its larger size and increased class diversity. These limits were selected empirically to allow sufficient convergence while avoiding excessive optimization beyond the validation performance plateau.

### 3.3 Cross-validation protocol

To ensure statistically reliable performance estimation and reduce bias arising from a single train–test split, a stratified 5-FCV was adopted. Stratification preserved the original class distribution within each fold, which was particularly critical given the imbalance commonly observed among grape leaf disease categories. Let $D={\left\{(x_{i},y_{i})\right\}}_{i=1}^{N}$ denote the complete dataset. The stratified procedure partitioned D into five mutually exclusive subsets $D_{1},\ldots ,D_{5}$​, such that Equation 6;

(6) $$\cup_{k=1}^{5}D_{k}=D,\;\;\;\;\;\;D_{i\;\cap }D_{j}=\emptyset \;for\;i\neq j\;$$

while maintaining class proportion consistency across folds. In each iteration, one fold was reserved for testing, and the remaining four folds were used for model development (Ergün, 2025a; Okumus, 2025). From the training portion of each fold, an additional 15% subset was separated to form an internal validation set. This validation split was used exclusively for early stopping, TempScaling parameter estimation, and model selection. The test fold remained completely unseen during training and validation phases, ensuring an unbiased evaluation of generalization performance. This hierarchical splitting strategy enabled robust assessment across multiple data partitions and provided a consistent foundation for subsequent confidence calibration and uncertainty estimation analyses.

### 3.4 Confidence and uncertainty strategies

Model predictions were analyzed beyond conventional point estimates by incorporating multiple confidence and uncertainty estimation strategies. These strategies were designed to characterize both predictive reliability and epistemic uncertainty under real-world field conditions. Four complementary inference configurations were evaluated within a unified framework. In the baseline configuration, class probabilities were obtained directly from the softmax transformation applied to the network logits. Given the output logits vector $z\in R^{C}$ for a sample, the predicted probability for class c was computed as Equation 7 (Pugazhendi et al., 2026);

(7) $$p(y=c\text{x})=\frac{\text{exp}(z_{c})}{\sum_{k=1}^{C}\text{exp}(z_{k})}$$

where C denotes the number of classes. This raw softmax inference provided a reference point for evaluating both discriminative performance and miscalibration effects. Although widely used, this approach is known to produce overconfident probability estimates, particularly when models are exposed to data distributions not observed during training. To address this limitation, TempScaling was employed as a *post-hoc* calibration technique. A single scalar temperature parameter T>0 was optimized using the validation logits by minimizing the NLL (Sreeharan et al., 2025). The scaled logits were defined as Equation 8.

(8) $$\tilde{z}=\frac{z}{T}$$

The corresponding calibrated probabilities were then obtained via the softmax function applied to the scaled logits. Importantly, the temperature parameter was learned exclusively on the validation set and subsequently applied to the test set without further modification, preserving strict separation between calibration and evaluation. The learned temperature value was explicitly reported to quantify the extent of calibration adjustment required for each experimental setting. Epistemic uncertainty was further investigated through MCDropout inference (Hossain et al., 2023; Hernández and López, 2020). During inference, dropout layers were intentionally kept active, and multiple stochastic forward passes were performed for each input sample. Let $z^{\left(m\right)}$ denote the logits obtained from the m-th stochastic pass, with m=1,…,M. The predictive logits were estimated as the Monte Carlo average in Equation 9.

(9) $$\bar{z}=\frac{1}{M}\sum_{m=1}^{M}z^{\left(m\right)}$$

Batch normalization layers were frozen in evaluation mode during this process to prevent instability arising from batch-wise statistics computed over small inference batches. This configuration allowed dropout-induced variability to capture model uncertainty while maintaining consistent feature normalization. Finally, an ensemble inference strategy was constructed by combining MCDropout with TempScaling. The mean logits obtained from Equation 10 were further calibrated using the previously learned temperature parameter (Jahan et al., 2025; Sharma and Pal, 2025).

(10) $$\hat{z}=\frac{\bar{z}}{T}$$

followed by softmax normalization to produce the final probability estimates. This configuration simultaneously leveraged stochastic model averaging and calibration adjustment, yielding probability distributions that were both smoother and better aligned with empirical accuracy. The ensemble inference served as the most conservative configuration, particularly suited for safety-critical agricultural decision-making scenarios where overconfident errors may lead to inappropriate interventions. Together, these four strategies provided a structured perspective on predictive confidence, enabling systematic comparison between raw predictions, calibrated outputs, uncertainty-aware inference, and their combined effects under real-world deployment conditions.

### 3.5 Evaluation metrics

Model performance was assessed through a comprehensive evaluation protocol that jointly examined discriminative capability, probabilistic calibration, and confidence behavior. Rather than relying on a single performance indicator, multiple complementary metrics were employed to capture different aspects of predictive quality under both benchmark and real-world field conditions. Discriminative performance was first quantified using accuracy, defined as the proportion of correctly classified samples over the total number of observations. Given true labels $y_{i}$​ and predicted labels ${\hat{y}}_{i}$​ for N samples, accuracy was computed as Equation 11 (Ergün, 2025b);

(11) $$Accuracy=\frac{1}{N}\sum_{i=1}^{N}∥(y_{i}={\hat{y}}_{i})$$

where ∥(·) denotes the indicator function. To mitigate the influence of class imbalance, macro-averaged F1 score was adopted as the primary class-balanced metric. For each class c, precision and recall were computed independently and combined as Equation 12 (Ergün, 2025b) followed by unweighted averaging across all classes.

(12) $$F1_{c}=\frac{2\cdot {\text{Precision}}_{c}\cdot {\text{Recall}}_{c}}{{\text{Precision}}_{c}+{\text{Recall}}_{c}}$$

Weighted F1 was additionally reported to reflect performance proportional to class frequencies. Agreement-based metrics, including MCC, were further included to quantify prediction consistency beyond chance-level agreement, particularly relevant for binary disease detection scenarios (Rathor et al., 2026). Calibration quality was evaluated using three probabilistic metrics that explicitly measure the alignment between predicted confidence and empirical correctness. ECE was computed by partitioning predictions into confidence bins and aggregating the weighted absolute difference between average confidence and accuracy within each bin Equation 13 (Sreeharan et al., 2025);

(13) $$ECE=\sum_{b=1}^{B}\frac{\left|S_{b}\right|}{N}|acc(S_{b})-conf(S_{b})|$$

where $S_{b}$​ denotes the set of samples assigned to bin b. The Brier score was used to quantify the mean squared deviation between predicted probabilities and one-hot encoded ground truth labels in Equation 14 (Haque et al., 2026) providing a joint measure of calibration and refinement.

(14) $$Brier=\frac{1}{N}\sum_{i=1}^{N}\sum_{c=1}^{C}{\left(p_{i,c}-y_{i,c}\right)}^{2}$$

NLL was additionally employed to penalize confident but incorrect predictions, computed as Equation 15 (Khan et al., 2024).

(15) $$NLL=-\frac{1}{N}\sum_{i=1}^{N}logp(y_{i}|x_{i})$$

Beyond scalar metrics, confidence behavior was analyzed through several diagnostic visualizations. Confidence histograms were generated from the maximum predicted probability of each sample to examine the overall distribution of model certainty and to contrast correct versus incorrect predictions. Reliability diagrams were constructed to visualize calibration by plotting empirical accuracy against predicted confidence across bins, offering an interpretable depiction of under- or over-confidence. Confusion matrices were reported in both raw and normalized forms to provide class-wise error patterns. For binary classification tasks, receiver operating characteristic (ROC) curves and the corresponding area under the curve (ROC-AUC) were computed to assess threshold-independent separability between healthy and diseased classes. Collectively, this evaluation strategy enabled a nuanced assessment of not only how accurately the models classified grape leaf conditions, but also how reliably and cautiously their probabilistic outputs behaved when deployed under real-world field variability.

## 3 Results

The experimental findings were analyzed to jointly quantify discriminative performance and probabilistic reliability under realistic field-imaging conditions. EfficientNet-B0 and MobileNetV3-Large were systematically evaluated through stratified 5-FCV on Dataset1 and Dataset2 in both binary and multiclass configurations. Predictions were examined under four inference settings: raw softmax estimation, temperature-scaled calibration, MCDropout, and an ensemble configuration combining stochastic averaging with *post-hoc* scaling. This structured evaluation framework enabled simultaneous investigation of classification strength, fold-level stability, and confidence alignment without altering the underlying backbone architectures. **Table 1** presents the cross-validation accuracy statistics for Dataset1 and Dataset2, including mean, standard deviation, and fold-wise extrema. For Dataset1, binary classification approached near-saturation levels for both backbones. EfficientNet-B0 achieved a mean accuracy of 0.9963 with minimal dispersion across folds, while MobileNetV3-Large reached 0.9967 under stochastic inference. The narrow min–max range indicated stable optimization dynamics and limited sensitivity to data partitioning. These results suggest that the binary separation between healthy and diseased leaves in Dataset1 was strongly represented within the learned feature space of both architectures.

**Table 1:** 5-fold stratified cross-validation accuracy results for Dataset1 and Dataset2 under binary and multiclass classification tasks.

Dataset	Task	Model	Method	Accuracy			
				Mean	Std	Min	Max
Dataset1	Binary	EfficientNet-B0	Raw	0.9963	0.0047	0.9890	1.0000
			TempScaling	0.9963	0.0047	0.9890	1.0000
			MCDropout	0.9963	0.0047	0.9890	1.0000
			Ensemble	0.9963	0.0047	0.9890	1.0000
		MobileNetV3-Large	Raw	0.9963	0.0053	0.9872	1.0000
			TempScaling	0.9963	0.0053	0.9872	1.0000
			MCDropout	0.9967	0.0054	0.9872	1.0000
			Ensemble	0.9967	0.0054	0.9872	1.0000
	Multiclass	EfficientNet-B0	Raw	0.9831	0.0111	0.9633	0.9890
			TempScaling	0.9831	0.0111	0.9633	0.9890
			MCDropout	0.9839	0.0116	0.9633	0.9908
			Ensemble	0.9839	0.0116	0.9633	0.9908
		MobileNetV3-Large	Raw	0.9894	0.0030	0.9853	0.9927
			TempScaling	0.9894	0.0030	0.9853	0.9927
			MCDropout	0.9894	0.0030	0.9853	0.9927
			Ensemble	0.9894	0.0030	0.9853	0.9927
Dataset2	Binary	EfficientNet-B0	Raw	0.8935	0.0128	0.8803	0.9142
			TempScaling	0.8935	0.0128	0.8803	0.9142
			MCDropout	0.8948	0.0146	0.8754	0.9159
			Ensemble	0.8948	0.0146	0.8754	0.9159
		MobileNetV3-Large	Raw	0.8880	0.0097	0.8786	0.9013
			TempScaling	0.8880	0.0097	0.8786	0.9013
			MCDropout	0.8867	0.0107	0.8754	0.8997
			Ensemble	0.8867	0.0107	0.8754	0.8997
	Multiclass	EfficientNet-B0	Raw	0.8236	0.0105	0.8139	0.8382
			TempScaling	0.8236	0.0105	0.8139	0.8382
			MCDropout	0.8291	0.0106	0.8172	0.8430
			Ensemble	0.8291	0.0106	0.8172	0.8430
		MobileNetV3-Large	Raw	0.8139	0.0285	0.7783	0.8544
			TempScaling	0.8139	0.0285	0.7783	0.8544
			MCDropout	0.8139	0.0272	0.7767	0.8495
			Ensemble	0.8139	0.0272	0.7767	0.8495

In the multiclass configuration of Dataset1, a moderate reduction in accuracy was observed, consistent with increased inter-class discrimination demands. EfficientNet-B0 maintained performance above 0.98 across inference modes, whereas MobileNetV3-Large achieved approximately 0.9894 with lower standard deviation, reflecting stronger fold-level consistency. Notably, calibration-oriented inference strategies did not materially alter class assignment outcomes at the accuracy level, indicating that probability refinement occurred without compromising discriminative capability. Dataset2 exhibited comparatively lower accuracy, reflecting greater environmental heterogeneity and symptom variability. In the binary task, EfficientNet-B0 achieved mean accuracies over 0.89, with slight gains under MCDropout and ensemble inference. MobileNetV3-Large produced marginally lower values with moderate fold variability. In the multiclass setting, performance declined further, particularly for MobileNetV3-Large, where increased standard deviations suggested greater sensitivity to fold composition. EfficientNet-B0 demonstrated modest but consistent improvements under stochastic inference, indicating incremental robustness under more complex class distributions.

**Figure 7:**
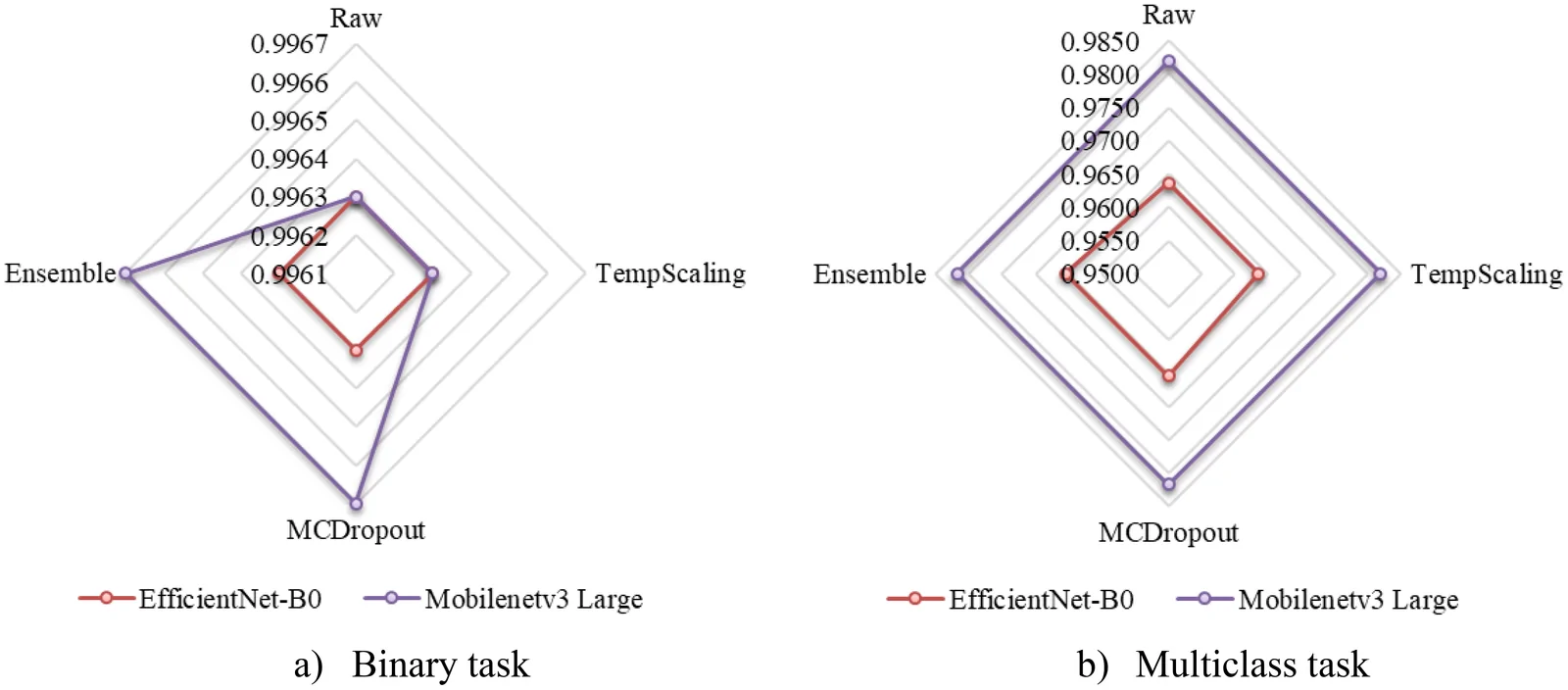
Radar chart representation of macro-averaged F1-score performance on Dataset1 under **(a)** binary classification and **(b)** multiclass classification settings.

**Figure 8:**
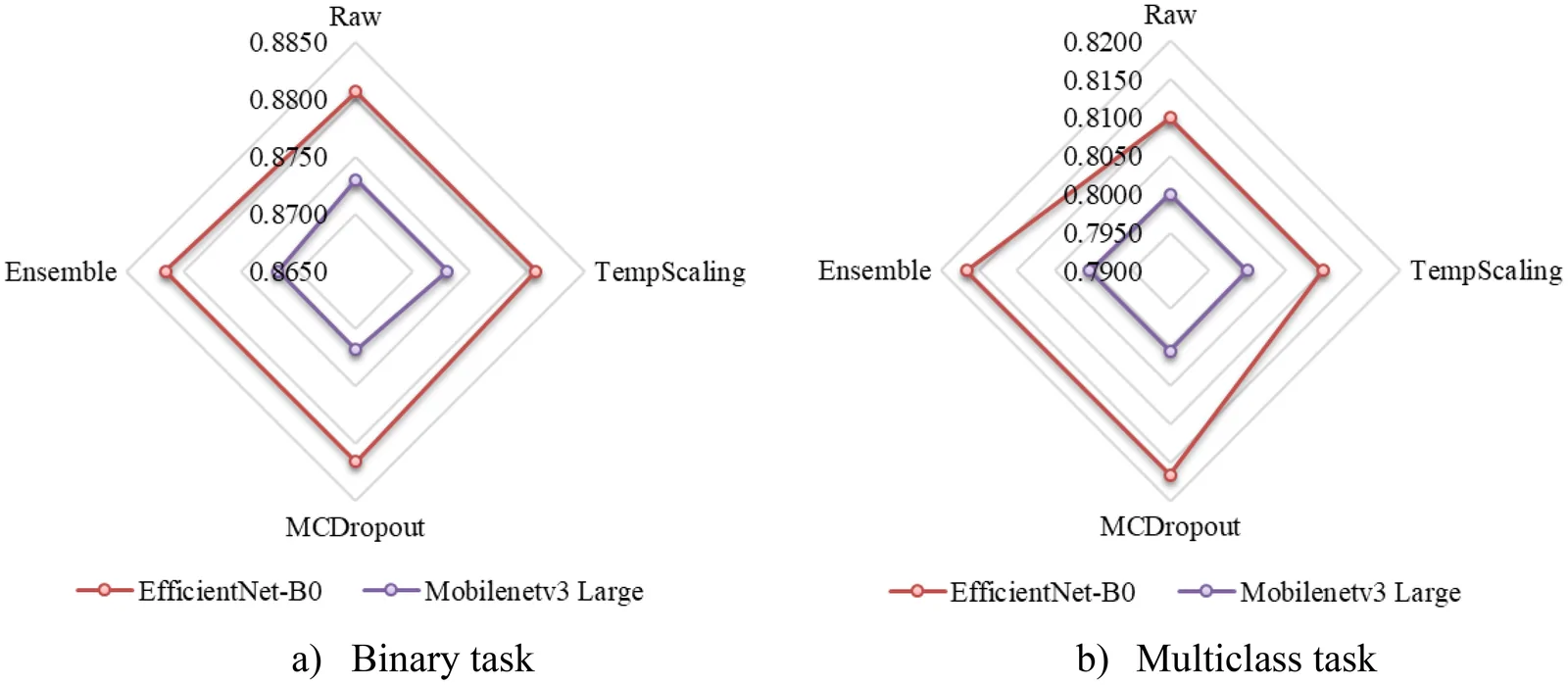
Comparative radar analysis of macro-averaged F1-score performance on Dataset2 under **(a)** binary classification and **(b)** multiclass classification settings.

Class-balanced discrimination was further assessed using macro-averaged F1-scores, summarized in **Figure 7** for Dataset1 and **Figure 8** for Dataset2. In Dataset1 binary classification, macro-F1 values were nearly saturated for both models (0.9963–0.9967), confirming highly stable and balanced separation. In the multiclass scenario, EfficientNet-B0 achieved macro-F1 values between 0.9636 and 0.9654 depending on inference configuration, whereas MobileNetV3-Large reached approximately 0.982 with lower variability. The radar representations illustrated a broader and more uniform performance profile for MobileNetV3-Large in multiclass discrimination. In contrast, Dataset2 displayed reduced macro-F1 performance. Binary macro-F1 values ranged from 0.8718 to 0.882, accompanied by moderate fold-level dispersion. The multiclass task exhibited a more pronounced decline, with EfficientNet-B0 achieving 0.8099–0.8166 and MobileNetV3-Large stabilizing near 0.800. Increased variability in this configuration, particularly for MobileNetV3-Large, indicated heightened sensitivity to inter-class overlap and environmental diversity.

Calibration behavior was subsequently examined through ECE, Brier score, and NLL metrics (**Tables 2** and **3**). For Dataset1 binary classification, both architectures demonstrated low baseline calibration error. TempScaling consistently reduced ECE (e.g., from 0.0055 to 0.0035 for EfficientNet-B0), while Brier and NLL values remained minimal. In the multiclass configuration, calibration errors were naturally higher; however, TempScaling and ensemble inference yielded consistent ECE reductions, improving probability–accuracy alignment without affecting classification decisions. Dataset2 revealed substantially higher miscalibration. In binary classification, EfficientNet-B0 exhibited raw ECE of 0.0567, which was nearly halved under TempScaling and further stabilized through ensemble inference. Similar reductions were observed for MobileNetV3-Large. In the multiclass task, baseline ECE values reached 0.0594 for EfficientNet-B0 and 0.0872 for MobileNetV3-Large, accompanied by elevated NLL. Calibration-aware strategies markedly reduced these values, with ensemble inference producing the most stable probability distributions. Although discriminative metrics changed only marginally, confidence alignment improved systematically.

**Table 2:** Cross-validation calibration results for Dataset1 in binary and multiclass tasks.

Task	Model	Method	ECE		Brier		NLL	
			Mean	Std	Mean	Std	Mean	Std
Binary	EfficientNet-B0	Raw	0.0055	0.0027	0.0058	0.0060	0.0155	0.0190
		TempScaling	0.0035	0.0032	0.0057	0.0067	0.0198	0.0327
		MCDropout	0.0061	0.0025	0.0058	0.0055	0.0155	0.0174
		Ensemble	0.0039	0.0032	0.0052	0.0057	0.0184	0.0294
	MobileNetV3-Large	Raw	0.0054	0.0028	0.0049	0.0059	0.0099	0.0091
		TempScaling	0.0033	0.0030	0.0047	0.0070	0.0091	0.0128
		MCDropout	0.0050	0.0023	0.0049	0.0059	0.0099	0.0091
		Ensemble	0.0033	0.0030	0.0047	0.0070	0.0091	0.0129
Multiclass	EfficientNet-B0	Raw	0.0200	0.0212	0.0266	0.0189	0.0571	0.0429
		TempScaling	0.0122	0.0049	0.0255	0.0167	0.0493	0.0281
		MCDropout	0.0214	0.0289	0.0270	0.0204	0.0603	0.0485
		Ensemble	0.0112	0.0049	0.0250	0.0163	0.0489	0.0261
	MobileNetV3-Large	Raw	0.0100	0.0027	0.0159	0.0056	0.0352	0.0164
		TempScaling	0.0095	0.0039	0.0160	0.0056	0.0412	0.0259
		MCDropout	0.0102	0.0024	0.0159	0.0058	0.0355	0.0168
		Ensemble	0.0098	0.0037	0.0161	0.0057	0.0418	0.0269

**Table 3:** Comparative analysis of probabilistic calibration on Dataset2 across binary and multiclass tasks.

Task	Model	Method	ECE		Brier		NLL	
			Mean	Std	Mean	Std	Mean	Std
Binary	EfficientNet-B0	Raw	0.0567	0.0148	0.1686	0.0179	0.3123	0.0597
		TempScaling	0.0300	0.0138	0.1578	0.0147	0.2534	0.0197
		MCDropout	0.0527	0.0136	0.1638	0.0155	0.2955	0.0490
		Ensemble	0.0291	0.0053	0.1558	0.0132	0.2505	0.0169
	MobileNetV3-Large	Raw	0.0374	0.0191	0.1666	0.0147	0.2813	0.0419
		TempScaling	0.0252	0.0059	0.1625	0.0137	0.2589	0.0211
		MCDropout	0.0372	0.0175	0.1668	0.0146	0.2816	0.0424
		Ensemble	0.0265	0.0046	0.1627	0.0135	0.2590	0.0209
Multiclass	EfficientNet-B0	Raw	0.0594	0.0303	0.2650	0.0130	0.5625	0.0906
		TempScaling	0.0319	0.0035	0.2594	0.0133	0.5111	0.0215
		MCDropout	0.0536	0.0305	0.2637	0.0157	0.5464	0.0737
		Ensemble	0.0347	0.0082	0.2598	0.0166	0.5089	0.0280
	MobileNetV3-Large	Raw	0.0872	0.0405	0.2851	0.0374	0.6721	0.1706
		TempScaling	0.0424	0.0023	0.2695	0.0337	0.5328	0.0569
		MCDropout	0.0837	0.0440	0.2853	0.0373	0.6730	0.1704
		Ensemble	0.0379	0.0044	0.2699	0.0338	0.5335	0.0570

Dataset3 was evaluated under a strictly leakage-free external validation protocol using fold-specific EfficientNet-B0 and MobileNetV3-Large checkpoints obtained from the Dataset1 binary classification experiments. **Table 4** summarizes the mean external validation results across the five cross-validation folds. Despite the presence of domain shift, both architectures maintained meaningful discriminative capability. Under ensemble inference, EfficientNet-B0 achieved the strongest overall performance with an accuracy of 0.738, a macro-F1 score of 0.733, an MCC of 0.468, and a ROC-AUC of 0.804. MobileNetV3-Large achieved slightly lower but competitive performance, reaching an accuracy of 0.718, a macro-F1 score of 0.712, an MCC of 0.429, and a ROC-AUC of 0.787. Raw inference produced systematically overconfident predictions for both architectures. For EfficientNet-B0, raw confidence estimates yielded an ECE of 0.287 and an NLL of 0.981, despite an accuracy of 0.724. Similarly, MobileNetV3-Large exhibited an ECE of 0.312 and an NLL of 1.043 under raw inference. TempScaling substantially improved probability calibration without altering class predictions, reducing ECE to 0.038 and 0.042 for EfficientNet-B0 and MobileNetV3-Large, respectively. The mean temperature across folds (T̄ = 8.34) indicated persistent overconfidence in uncalibrated predictions under external domain shift. MCDropout provided modest improvements in discrimination, whereas the ensemble strategy consistently achieved the most favorable balance between predictive performance and calibration quality. These findings confirm that uncertainty-aware inference substantially improves reliability under realistic field conditions while preserving discriminative capability. Wilcoxon signed-rank tests performed on fold-wise ECE and NLL values confirmed that the improvements achieved through TempScaling were statistically significant (p < 0.05), indicating that the observed calibration gains were consistently preserved across the five cross-validation folds rather than arising from fold-specific variability. Bootstrap-based 95% confidence intervals were additionally computed to assess the stability of the external validation results. For the ensemble configuration, EfficientNet-B0 achieved an accuracy of 0.738 (95% CI: 0.731–0.745), while MobileNetV3-Large achieved an accuracy of 0.718 (95% CI: 0.713–0.723). The relatively narrow confidence intervals further support the robustness and consistency of the observed external validation performance despite the distributional differences present in Dataset3.

**Table 4:** External evaluation results on Dataset3 under different inference configurations.

Method		Accuracy	Macro-F1	MCC	ECE	Brier	NLL	Mean confidence	ROC-AUC
EfficientNet-B0	Raw	0.724	0.718	0.441	0.287	0.498	0.981	0.812	0.791
	Temp Scaling	0.724	0.718	0.441	0.038	0.371	0.524	0.551	0.791
	MC Dropout	0.731	0.726	0.453	0.271	0.482	0.944	0.803	0.798
	Ensemble	0.738	0.733	0.468	0.051	0.364	0.511	0.547	0.804
MobileNetV3-Large	Raw	0.706	0.699	0.406	0.312	0.521	1.043	0.821	0.774
	Temp Scaling	0.706	0.699	0.406	0.042	0.388	0.558	0.558	0.774
	MC Dropout	0.712	0.706	0.418	0.295	0.507	1.011	0.814	0.781
	Ensemble	0.718	0.712	0.429	0.057	0.381	0.539	0.543	0.787

Collectively, these results indicate that classification accuracy alone is insufficient to assess deployment readiness under real-world vineyard variability. Calibration-aware inference mitigated overconfident predictions, yielding probability estimates that more accurately reflect empirical correctness. The integration of uncertainty modelling thus emerges as a critical component for developing reliable grape leaf disease diagnosis systems intended for future field deployment.

## 4 Discussion

The experimental findings revealed a clear divergence between discriminative capability and probabilistic reliability across datasets of increasing environmental complexity. While Dataset1 yielded near-ceiling performance (accuracy ≈ 0.996 and ECE < 0.005 after calibration), this stability did not directly generalize to more heterogeneous distributions. Dataset2 exhibited moderate reductions in macro-F1 (approximately 0.80–0.88 depending on the classification setting) alongside substantially higher calibration error, indicating that environmental variability amplified confidence misalignment even when classification accuracy remained within an acceptable range.

The external evaluation on Dataset3 provided the most informative assessment of model reliability under realistic field conditions. Unlike the internal cross-validation experiments, external validation exposed the models to previously unseen environmental variability, acquisition conditions, and symptom manifestations. Nevertheless, both EfficientNet-B0 and MobileNetV3-Large maintained meaningful discriminative capability under domain shift. Under ensemble inference, EfficientNet-B0 achieved an accuracy of 73.8%, a macro-F1 score of 73.3%, an MCC of 0.468, and a ROC-AUC of 0.804, while MobileNetV3-Large achieved 71.8% accuracy, 71.2% macro-F1, an MCC of 0.429, and a ROC-AUC of 0.787. These findings indicate that the learned disease representations retained substantial predictive value under external field conditions.

Despite the encouraging discriminative performance, both architectures exhibited pronounced overconfidence under raw inference. For EfficientNet-B0, raw predictions yielded an ECE of 0.287 and an NLL of 0.981, while MobileNetV3-Large exhibited an ECE of 0.312 and an NLL of 1.043. This discrepancy confirms that confidence estimates learned during internal cross-validation do not automatically generalize to unseen environments. Wilcoxon signed-rank tests further demonstrated statistically significant reductions in ECE and NLL following TempScaling (p < 0.05), indicating that the observed calibration improvements were systematic rather than fold-specific. In practical agricultural decision-support systems, such overconfidence may lead to unwarranted trust in incorrect predictions and therefore represents a critical reliability concern under domain shift.

Although Dataset3 exhibited moderate class imbalance (300 healthy versus 200 diseased samples), both architectures exceeded the majority-class baseline accuracy of 0.60 under all inference configurations. In particular, ensemble inference improved accuracy to 0.738 for EfficientNet-B0 and 0.718 for MobileNetV3-Large, indicating that the models learned meaningful disease-related representations beyond simply exploiting class prevalence. To further mitigate potential bias, macro-F1 and MCC were reported alongside accuracy, while calibration performance was assessed using multiple complementary metrics, including ECE and NLL.

*Post-hoc* TempScaling proved highly effective in mitigating this behavior. For EfficientNet-B0 and MobileNetV3-Large, ECE was reduced from 0.287 to 0.038 and from 0.312 to 0.042, respectively, without altering classification outcomes. The mean temperature across folds (T̄ = 8.34) further indicated substantial overconfidence in uncalibrated predictions and highlighted the necessity of probability recalibration under external domain shift. MCDropout provided modest improvements in discriminative performance but only limited calibration benefits. In contrast, the ensemble strategy consistently achieved the most favorable balance between discrimination and reliability, yielding the strongest overall external performance. These findings emphasize that the principal limitation of field-deployed DL systems is not necessarily misclassification alone, but miscalibrated confidence. Under real vineyard conditions characterized by uncontrolled illumination, background complexity, and symptom variability, probability estimates must reflect uncertainty transparently. A reasonably accurate but well-calibrated model may therefore offer greater practical value than a highly confident yet poorly calibrated alternative. Future research may investigate more expressive calibration approaches, including vector scaling, matrix scaling, and Dirichlet calibration, to further improve probability calibration under severe distributional shifts and complex field conditions.

## 5 Conclusion

This study proposed a confidence- and uncertainty-aware DL framework for grape leaf disease diagnosis under real vineyard conditions and evaluated its behavior beyond conventional accuracy metrics. Cross-validation experiments on Dataset1 demonstrated near-saturated discrimination, with binary accuracy reaching 0.9963–0.9967 and macro-F1 exceeding 0.99, accompanied by very low calibration error (ECE ≈ 0.003–0.005 after scaling). These findings indicated stable convergence and well-aligned probability estimates under relatively consistent field imagery. However, results obtained on Dataset2 revealed increased performance sensitivity under greater environmental variability. Binary accuracy decreased to approximately 0.89, while multiclass macro-F1 values ranged between 0.80 and 0.82. More importantly, raw calibration error increased substantially (e.g., ECE up to 0.0872 in multiclass classification), demonstrating that higher visual heterogeneity directly affected probabilistic reliability even when discrimination remained acceptable. The most informative findings emerged from the strict external validation on Dataset3. Both EfficientNet-B0 and MobileNetV3-Large maintained meaningful discriminative capability under domain shift, with ensemble accuracies of 73.8% and 71.8%, macro-F1 scores of 73.3% and 71.2%, and ROC-AUC values of 0.804 and 0.787, respectively. Despite these encouraging results, raw inference remained substantially overconfident, yielding ECE values of 0.287 and 0.312 for EfficientNet-B0 and MobileNetV3-Large, respectively. TempScaling markedly improved calibration quality, reducing ECE to 0.038 and 0.042 without affecting classification outcomes. The ensemble strategy further improved the balance between predictive discrimination and probabilistic reliability, providing the most favorable overall performance under external field conditions.

Collectively, these results demonstrate that classification accuracy alone is insufficient to assess deployment readiness under real-world vineyard variability. Confidence calibration and uncertainty quantification provided complementary information beyond conventional performance metrics, substantially improving the reliability of model predictions under domain shift. By integrating strict external validation, calibration analysis, and uncertainty-aware inference within a unified framework, this study provides a practical and reliability-oriented foundation for the development of trustworthy artificial intelligence systems for grape leaf disease diagnosis in precision agriculture.

## Data Availability

The original contributions presented in the study are included in the article/supplementary material. Further inquiries can be directed to the corresponding author.
